# Supplementary material for: High‐affinity interactions and signal transduction between Aβ oligomers and TREM2
Source: EMBO Mol Med. 2018 Oct 19;10(11):e9027. doi: 10.15252/emmm.201809027 (PMC6220267; doi:10.15252/emmm.201809027)
Supplement: Supplementary file 1 — Appendix [file EMMM-10-e9027-s001.pdf]

Appendix

Complete statistical results from figures 2-4-5-EV3.

Table of Contents:

Appendix Table S1. Statistical results for fig 2……………………………………………………………………………p2

Appendix Table S2. Statistical results for fig 4……………………………………………………………………………p3

Appendix Table S3. Statistical results for fig 5…………………………………………………………………….……….p4

Appendix Table S4. Statistical results for fig EV3……………………………………………………………………….p5

Appendix Table S1. Statistical results for fig 2.

**Figure 2**			
Tukey's multiple comparisons test	Significant?	Summary	Adjusted P Value
**TREM2 vs. R47H**	**Yes**	**#**	**0.0354**
**TREM2 vs. R62H**	**Yes**	**#**	**0.0481**
R47H vs. R62H	No	ns	0.6233

Appendix Table S2. Statistical results for fig 4.

Figure 4A

Bonferroni's multiple comparisons test	Significant?	Summary	Adjusted P Value
0			
mock vs. Fc	No	ns	>0.9999
sTREM2-Fc vs. Fc	No	ns	>0.9999
sTREM2 R47H-Fc vs. Fc	No	ns	>0.9999
sTREM2 R62H-Fc vs. Fc	No	ns	>0.9999
sTrem2-Fc vs. Fc	No	ns	>0.9999
100			
mock vs. Fc	No	ns	>0.9999
sTREM2-Fc vs. Fc	No	ns	>0.9999
sTREM2 R47H-Fc vs. Fc	No	ns	>0.9999
sTREM2 R62H-Fc vs. Fc	No	ns	>0.9999
sTrem2-Fc vs. Fc	No	ns	>0.9999
1000			
mock vs. Fc	No	ns	>0.9999
**sTREM2-Fc vs. Fc**	**Yes**	**	**0.0056**
**sTREM2 R47H-Fc vs. Fc**	**Yes**	*	**0.0168**
**sTREM2 R62H-Fc vs. Fc**	**Yes**	**	**0.0019**
**sTrem2-Fc vs. Fc**	**Yes**	***	**0.0003**
10000			
mock vs. Fc	No	ns	>0.9999
**sTREM2-Fc vs. Fc**	**Yes**	****	**<0.0001**
**sTREM2 R47H-Fc vs. Fc**	**Yes**	****	**<0.0001**
**sTREM2 R62H-Fc vs. Fc**	**Yes**	****	**<0.0001**
**sTrem2-Fc vs. Fc**	**Yes**	****	**<0.0001**

Figure 4B

Bonferroni's multiple comparisons test	Significant?	Summary	Adjusted P Value
mock vs. Fc	No	ns	>0.9999
**sTREM2-Fc vs. Fc**	**Yes**	****	**<0.0001**
sTREM1-Fc vs. Fc	No	ns	>0.9999
**sTREML1-Fc vs. Fc**	**Yes**	*	**0.0337**

Appendix Table S3. Statistical results for fig 5.

**Figure 5A: compared to TREM2**			
Bonferroni's multiple comparisons test	Significant?	Summary	Adjusted P Value
0			
TREM2 vs. mock	No	ns	>0.9999
TREM2 vs. TREM2 R47H	No	ns	>0.9999
0.01			
TREM2 vs. mock	No	ns	>0.9999
TREM2 vs. TREM2 R47H	No	ns	>0.9999
0.032			
TREM2 vs. mock	No	ns	>0.9999
TREM2 vs. TREM2 R47H	No	ns	>0.9999
0.1			
TREM2 vs. mock	No	ns	>0.9999
TREM2 vs. TREM2 R47H	No	ns	>0.9999
0.32			
TREM2 vs. mock	No	ns	0.3875
TREM2 vs. TREM2 R47H	No	ns	0.8168
1			
**TREM2 vs. mock**	**Yes**	*****	**0.0435**
TREM2 vs. TREM2 R47H	No	ns	>0.9999
3.16			
**TREM2 vs. mock**	**Yes**	********	**<0.0001**
**TREM2 vs. TREM2 R47H**	**Yes**	**#**	**0.0319**
10			
**TREM2 vs. mock**	**Yes**	********	**<0.0001**
**TREM2 vs. TREM2 R47H**	**Yes**	**####**	**<0.0001**

**Figure 5A: compared to mock**			
Bonferroni's multiple comparisons test	Significant?	Summary	Adjusted P Value
0			
mock vs. TREM2	No	ns	>0.9999
mock vs. TREM2 R47H	No	ns	>0.9999
0.01			
mock vs. TREM2	No	ns	>0.9999
mock vs. TREM2 R47H	No	ns	>0.9999
0.032			
mock vs. TREM2	No	ns	>0.9999
mock vs. TREM2 R47H	No	ns	>0.9999
0.1			
mock vs. TREM2	No	ns	>0.9999
mock vs. TREM2 R47H	No	ns	>0.9999
0.32			

mock vs. TREM2	No	ns	0.3875
mock vs. TREM2 R47H	No	ns	>0.9999
1			
**mock vs. TREM2**	**Yes**	*	**0.0435**
mock vs. TREM2 R47H	No	ns	>0.9999
3.16			
**mock vs. TREM2**	**Yes**	****	**<0.0001**
mock vs. TREM2 R47H	No	ns	0.0767
10			
**mock vs. TREM2**	**Yes**	****	**<0.0001**
**mock vs. TREM2 R47H**	**Yes**	****	**<0.0001**

Figure 5B: compared to TREM2

Dunnett's multiple comparisons test	Significant?	Summary	Adjusted P Value
**CTL vs. TREM2**	**Yes**	****	**<0.0001**
**TREM2 R47H vs. TREM2**	**Yes**	*	**0.0124**
**TREM2 R62H vs. TREM2**	**Yes**	**	**0.0048**
TREM2 T96K vs. TREM2	No	ns	0.2604

Figure 5B: compared to TREM2 T96K

Dunnett's multiple comparisons test	Significant?	Summary	Adjusted P Value
**CTL vs. TREM2 T96K**	**Yes**	**####**	**<0.0001**
TREM2 vs. TREM2 T96K	No	ns	0.2604
**TREM2 R47H vs. TREM2 T96K**	**Yes**	**##**	**0.0012**
**TREM2 R62H vs. TREM2 T96K**	**Yes**	**###**	**0.0006**

Figure 5C: compared to mock

Tukey's multiple comparisons test	Significant?	Summary	Adjusted P Value
**mock vs. TREM2**	**Yes**	****	**<0.0001**
**mock vs. TREM2 R47H**	**Yes**	****	**<0.0001**
**mock vs. TREM2 R62H**	**Yes**	***	**0.0002**
**TREM2 vs. TREM2 R47H**	**Yes**	**#**	**0.0212**
**TREM2 vs. TREM2 R62H**	**Yes**	**##**	**0.0026**
TREM2 R47H vs. TREM2 R62H	No	ns	0.8189

Appendix Table S4. Statistical results for fig EV3.

**Figure EV3B**			
Bonferroni's multiple comparisons test	Significant?	Summary	Adjusted P Value
control			
Fc vs. control	No	ns	>0.9999
Fc vs. sTREM2-Fc	No	ns	>0.9999
Fc vs. sTREM2 R47H-Fc	No	ns	>0.9999
Fc vs. sTREM2 R62H-Fc	No	ns	>0.9999
Fc vs. sTrem2-Fc	No	ns	>0.9999
Ab42 fibrils			
Fc vs. control	No	ns	>0.9999
Fc vs. sTREM2-Fc	No	ns	>0.9999
Fc vs. sTREM2 R47H-Fc	No	ns	>0.9999
Fc vs. sTREM2 R62H-Fc	No	ns	>0.9999
Fc vs. sTrem2-Fc	No	ns	>0.9999
Ab42 monomers			
Fc vs. control	No	ns	>0.9999
Fc vs. sTREM2-Fc	No	ns	0.8033
Fc vs. sTREM2 R47H-Fc	No	ns	>0.9999
Fc vs. sTREM2 R62H-Fc	No	ns	0.957
Fc vs. sTrem2-Fc	No	ns	>0.9999
Ab42 oligomers			
Fc vs. control	No	ns	>0.9999
**Fc vs. sTREM2-Fc**	**Yes**	****	**<0.0001**
**Fc vs. sTREM2 R47H-Fc**	**Yes**	****	**<0.0001**
**Fc vs. sTREM2 R62H-Fc**	**Yes**	****	**<0.0001**
**Fc vs. sTrem2-Fc**	**Yes**	**	**0.0014**
