# Supplementary material for: High‐affinity interactions and signal transduction between Aβ oligomers and TREM2
Source: EMBO Mol Med. 2018 Oct 19;10(11):e9027. doi: 10.15252/emmm.201809027 (PMC6220267; doi:10.15252/emmm.201809027)
Supplement: Supplementary file 2 — Expanded View Figures PDF [file EMMM-10-e9027-s002.pdf]

Expanded View Figures

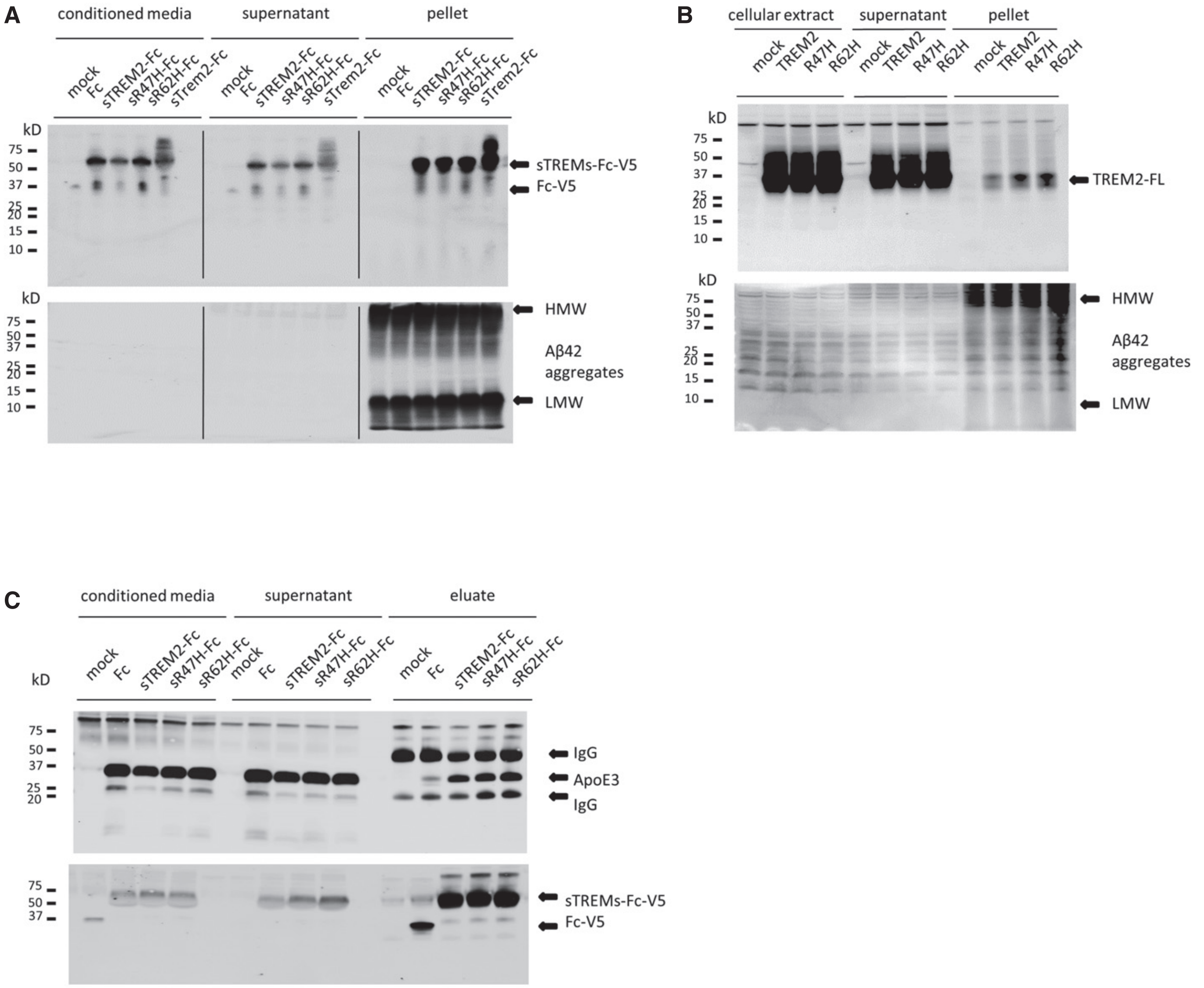

Figure EV1. Original uncropped images of the Western blots in Fig 1 showing the supernatant fractions.

A Original Western blot from Fig 1A.
B Original Western blot from Fig 1B.
C Original Western blot from Fig 1C.

© 2018 The Authors

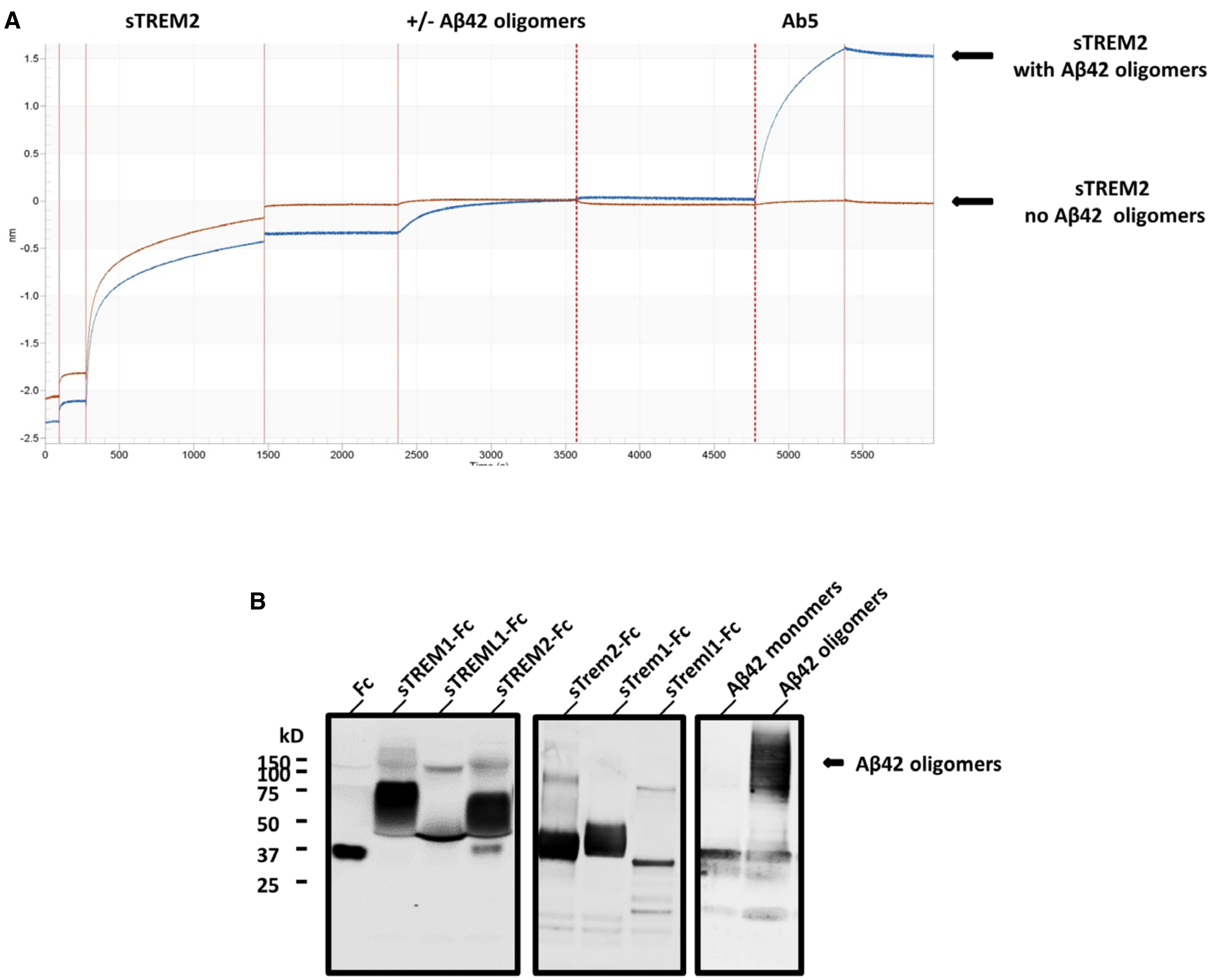

Figure EV2. Complementary experiments for the BioLayer Interferometry results in Fig 2.

A Schematic representation an Aβ antibody association on soluble TREM2-Fc pre-incubated with Aβ42 oligomers.

B Western blot analysis of purified soluble TREM-Fc and soluble trem2-Fc family members and Aβ oligomers.

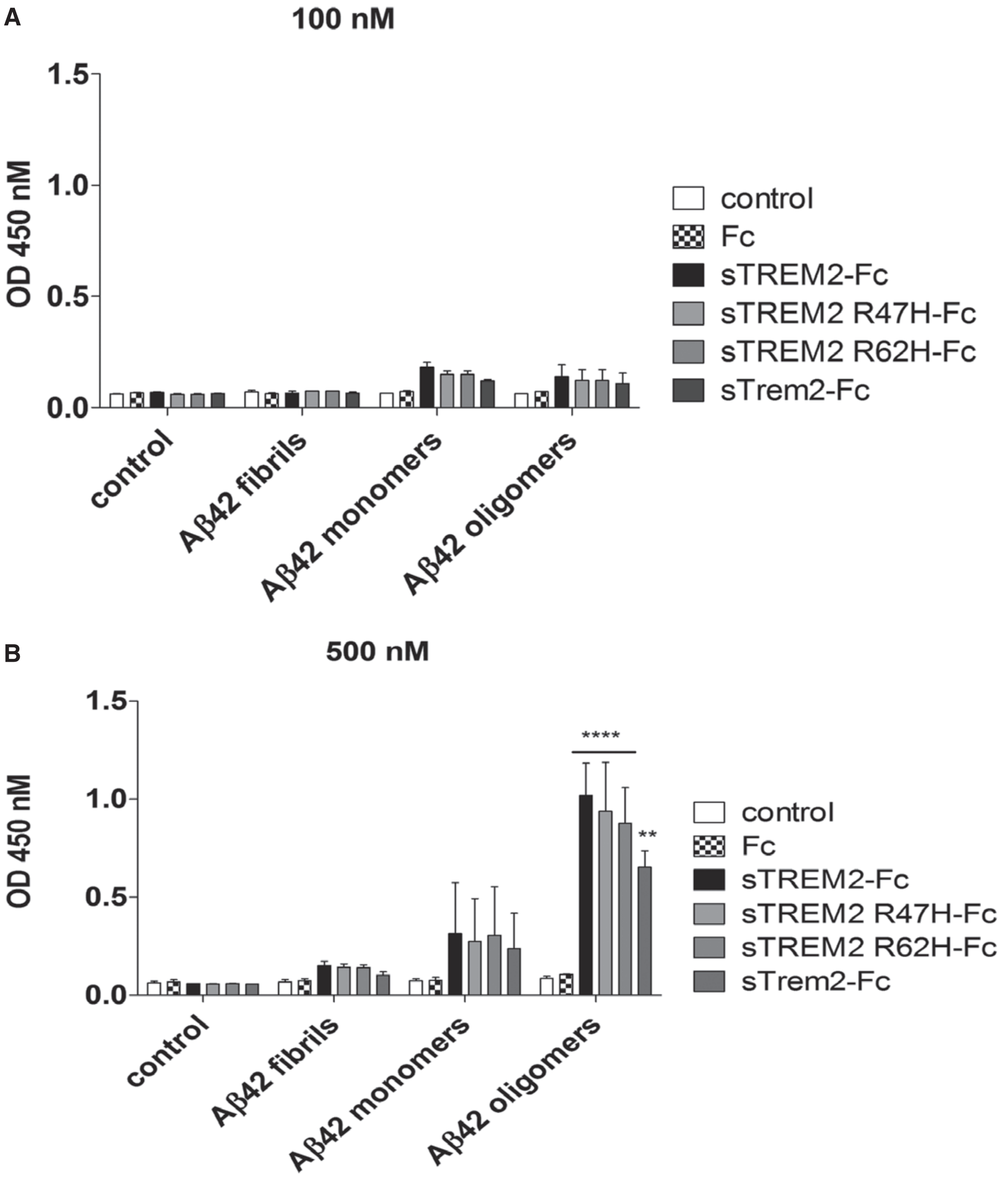

Figure EV3. Aβ42 fibrils failed to bind soluble TREM2-Fc measured by ELISA.

A, B ELISA plates coated with soluble TREM2-Fc and AD variants were incubated with various concentrations of Aβ42 aggregates or monomers or oligomers: (A) 100 nM and (B) 500 nM. Results were expressed as the OD450 averaged ± standard error ($n = 2$, $^{**}P < 0.01$, $^{****}P < 0.0001$, two-way ANOVA, Bonferroni multiple comparisons, compared to Fc, see Appendix Table S4 for exact P-values).

 © 2018 The Authors
