# Supplementary figures and images for: High‐affinity interactions and signal transduction between Aβ oligomers and TREM2
Source: EMBO Mol Med. 2018 Oct 19;10(11):e9027. doi: 10.15252/emmm.201809027 (PMC6220267; doi:10.15252/emmm.201809027)

A

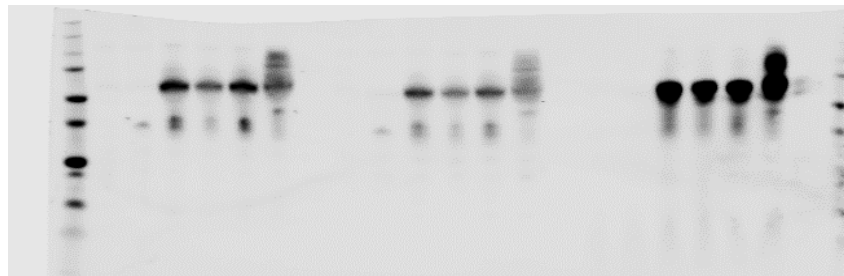

B

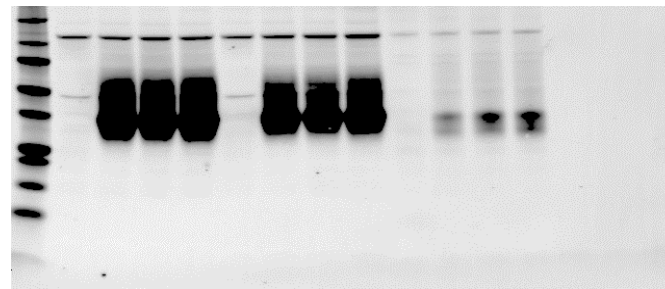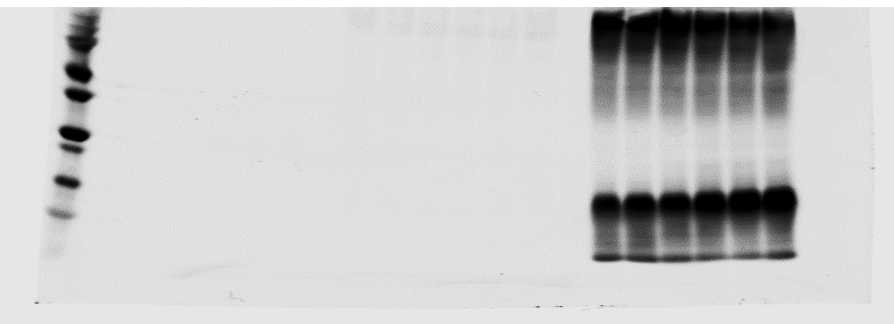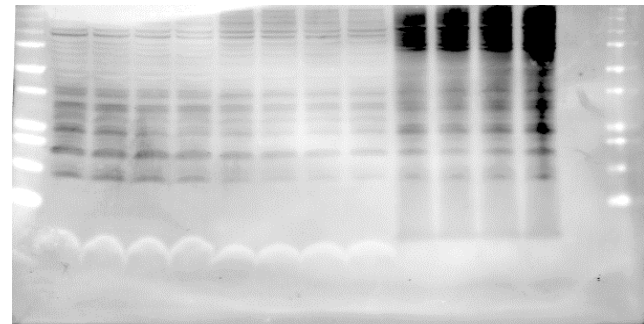

C

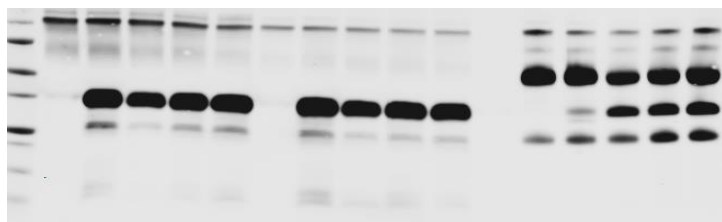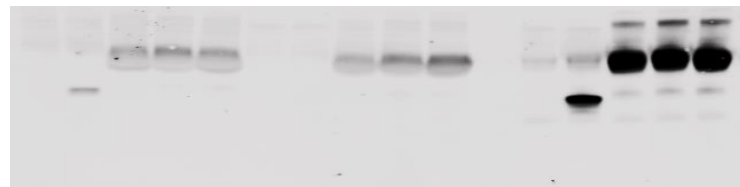

D

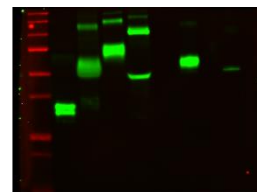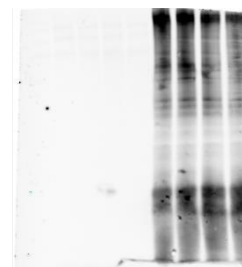

E

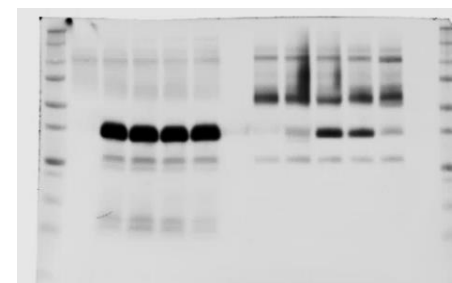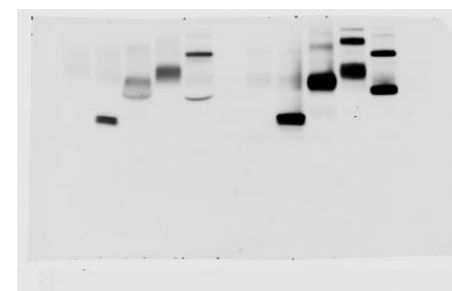

Original scan for Figure 1

Supplement: Supplementary file 4 — Source Data for Figure 1 [file EMMM-10-e9027-s003.pdf]

# Original scan for Figure 5

Fig 5D

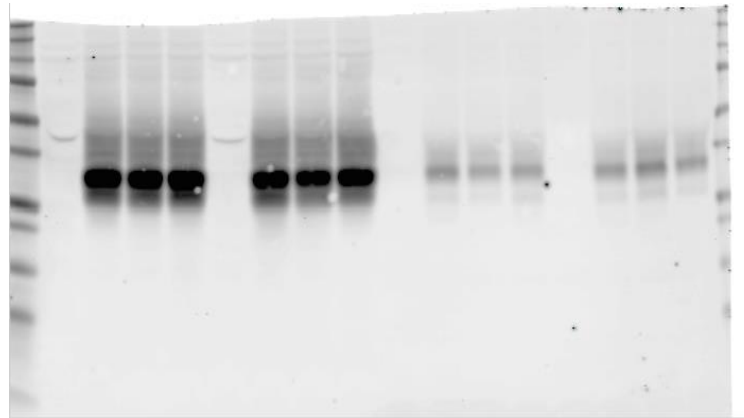

Fig5E

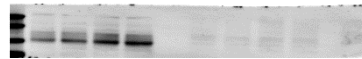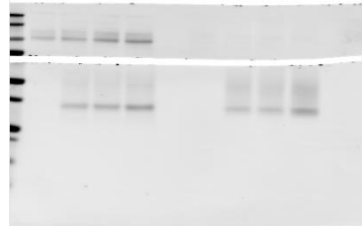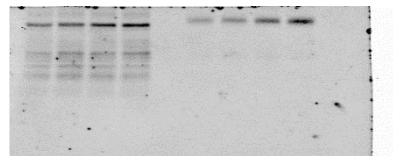

Supplement: Supplementary file 5 — Source Data for Figure 5 [file EMMM-10-e9027-s004.pdf]
